# Human umbilical cord mesenchymal stem cell-derived exosomal miR-27b attenuates subretinal fibrosis via suppressing epithelial–mesenchymal transition by targeting HOXC6

**DOI:** 10.1186/s13287-020-02064-0

**Published:** 2021-01-07

**Authors:** Dongli Li, Junxiu Zhang, Zijia Liu, Yuanyuan Gong, Zhi Zheng

**Affiliations:** 1 Department of Ophthalmology, Shanghai General Hospital, Shanghai Jiao Tong University School of Medicine, Shanghai, 20 080 China; 2 National Clinical Research Center for Eye Diseases, Shanghai, 200080 China; 3 Shanghai Key Laboratory of Ocular Fundus Diseases, Shanghai, 200080 China; 4 Shanghai Engineering Center for Visual Science and Photomedicine, NO.100, Haining Road, Hongkou District, Shanghai, 200080 China

**Keywords:** Epithelial–mesenchymal transition, Exosomes, Subretinal fibrosis, Mesenchymal stem cells

## Abstract

**Background and aim:**

Subretinal fibrosis resulting from neovascular age-related macular degeneration (nAMD) is one of the major causes of serious and irreversible vision loss worldwide, and no definite and effective treatment exists currently. Retinal pigmented epithelium (RPE) cells are crucial in maintaining the visual function of normal eyes and its epithelial–mesenchymal transition (EMT) is associated with the pathogenesis of subretinal fibrosis. Stem cell-derived exosomes have been reported to play a crucial role in tissue fibrosis by transferring their molecular contents. This study aimed to explore the effects of human umbilical cord-derived mesenchymal stem cell exosomes (hucMSC-Exo) on subretinal fibrosis in vivo and in vitro and to investigate the anti-fibrotic mechanism of action of hucMSC-Exo.

**Methods:**

In this study, human umbilical cord-derived mesenchymal stem cells (hucMSCs) were successfully cultured and identified, and exosomes were isolated from the supernatant by ultracentrifugation. A laser-induced choroidal neovascularization (CNV) and subretinal fibrosis model indicated that the intravitreal administration of hucMSC-Exo effectively alleviated subretinal fibrosis in vivo. Furthermore, hucMSC-Exo could efficaciously suppress the migration of retinal pigmented epithelial (RPE) cells and promote the mesenchymal–epithelial transition by delivering miR-27b-3p. The latent binding of miR-27b-3p to homeobox protein Hox-C6 (HOXC6) was analyzed by bioinformatics prediction and luciferase reporter assays.

**Results:**

This study showed that the intravitreal injection of hucMSC-Exo effectively ameliorated laser-induced CNV and subretinal fibrosis via the suppression of epithelial–mesenchymal transition (EMT) process. In addition, hucMSC-Exo containing miR-27b repressed the EMT process in RPE cells induced by transforming growth factor-beta2 (TGF-β2) via inhibiting HOXC6 expression.

**Conclusions:**

The present study showed that HucMSC-derived exosomal miR-27b could reverse the process of EMT induced by TGF-β2 via inhibiting HOXC6, indicating that the exosomal miR-27b/HOXC6 axis might play a vital role in ameliorating subretinal fibrosis. The present study proposed a promising therapeutic agent for treating ocular fibrotic diseases and provided insights into the mechanism of action of hucMSC-Exo on subretinal fibrosis.

## Introduction

Subretinal fibrosis is a wound-healing response generated against choroidal neovascularization (CNV) in neovascular age-related degeneration (nAMD) responsible for severe blindness worldwide [1]. In nAMD, CNV could stretch into Bruch’s membrane or the retinal pigmented epithelium (RPE) layer and subsequently develop into a fibrovascular membrane, leading to retinal edema and subretinal hemorrhage and hence causing the destruction of photoreceptors and RPE cells [2]. RPE cells are the main contributor to fibrotic scars at the end of nAMD. During subretinal fibrosis secondary to neovascular AMD, RPE cells lose their characteristic epithelial morphology and function and then transform into myofibroblasts, which is called epithelial–mesenchymal transition (EMT). EMT is considered to be a key feature of pathological tissue that needs to be repaired in subretinal fibrosis [3].

Currently, the standard therapeutic modalities for nAMD are anti-vascular endothelial growth factor (VEGF) treatment. However, in the long run, frequent intravitreal administration of anti-VEGF drugs is not always able to successfully suppress the growth of CNV, and its original efficacy gradually decreases over time [4, 5]. Several studies explained that anti-VEGFA treatment promoted the formation of fibrotic scars in the subretinal space, and fibrotic changes could reduce the efficacy of the anti-VEGFA pathway [6–8]. Therefore, the treatment of progressive fibrosis, including the reversal of EMT and the restoration of the epithelial phenotype, should be potential targets for neovascular AMD-related subretinal fibrosis [9, 10].

Mesenchymal stem cells (MSCs) are one of the most widely used stem cell types for immunomodulation, organ reconstruction, and tissue repair in clinical practice [11]. Their leading role in tissue repair is mediated by paracrine action [12]. Among these paracrine factors, exosomes are considered to be a novel treatment tool for delivering functional proteins, mRNA, miRNA, and lncRNA [13]. They are small membrane-bound particles and may serve as a strong candidate for cell-free therapies on account of overcoming the limitations of cell-based therapy while maintaining the advantages of their original cells [14]. In addition, exosomes are also ideal carriers and play a crucial role in intercellular communication because the exosomal membrane can protect their molecular content from degradation before reaching the target cells [15].

An increasing number of studies showed that exosomes secreted from MSCs could elicit significant therapeutic effects by suppressing fibrosis and improving their function in multiple-organ fibrosis models, such as liver, kidney, myocardium, and several retinal injury models [16–23]. In addition, studies also reported that the intravitreal injection of MSC-derived exosomes could ameliorate retinal laser injury by reducing damage and inhibiting apoptosis and inflammatory response [24]. Treatment with MSC-derived exosomes retarded the growth of CNV and decreased the number of fibroblasts and collagen fibers after laser photocoagulation, indicating that MSC-derived exosomes had great potential to reduce collagen formation in subretinal fibrosis [19]. Moreover, Mathew et al. proved that MSC-derived exosomes with an intravitreal injection could be maintained in the vitreous humor for more than 4 weeks, combined with the vitreous proteins in a dose-dependent manner, indicating that the administration of MSC-exosomes had a prolonged therapeutic effect and required fewer injections [22]. Based on the aforementioned findings, it was believed that human umbilical cord-derived mesenchymal stem cell exosomes (hucMSC-Exo) could serve as a cell-free regenerative therapy with great potential for treating subretinal fibrosis.

## Materials and methods

### Ethics statement

All experiments on animals were approved by the Animal Care Committee of Shanghai General Hospital. Principles of animal research abided by the guidelines of ARVO Statement for the Use of Animals in Ophthalmic and Vision Research. The study was sanctioned by the Ethics Committee of the Shanghai General Hospital (Approval No.2020SQ120), and informed consent was acquired before specimen collection.

### Cell culture

Human skin fibroblasts (HSFs) and ARPE19 cells were all purchased from the American Type Culture Collection (USA). The cells were maintained in Dulbecco’s modified Eagle’s medium (DMEM) and DMEM/Ham’s F-12 medium (DMEM/F12, Gibco, USA), respectively. Further, 10% fetal bovine serum (FBS) (Gibco) and 1% penicillin/streptomycin were supplemented with cells at 37 °C with 5% CO_2_.

### Preparation of hucMSCs

Briefly, fresh umbilical cords were obtained and washed with phosphate-buffered saline (PBS) containing 1% penicillin and streptomycin. Then, the blood vessels were taken out, and the remaining tissue was cut into 1-mm^3^ pieces on culture plates. The medium was replaced every 3 days. Nonadherent fragments were discarded, and the adherent cells were subcultured with 0.25% trypsin. The hucMSCs in passages 3–7 were used for further experiments.

The immune phenotype of hucMSCs was characterized by flow cytometry using a human MSC analysis kit (562245; BD Biosciences, USA), which contained four positive markers (FITC-CD90, PerCP Cy5.5-CD105, APC-CD73, and PE-CD44), five negative cocktails (PE-CD45, PE-CD34, PE-CD11b, PE-CD19, and PE-HLA-DR), and the respective isotype controls. The cells at a concentration of 5 × 10^6^ cells/mL in PBS were stained following the BD protocol. The analysis was carried out in a flow cytometer (Beckman Coulter, USA). Passage 3 hucMSCs were cultured in OriCell osteogenic and adipogenic differentiation media (Cyagen, Guangzhou, China) following the manufacturer’s protocol to identify the differentiation properties. After culturing for 23 days, hucMSCs were fixed and dyed with Alizarin red for osteogenic cells and Oil red for adipose cells. The cells cultured in a normal medium served as controls.

### Extraction and identification of hucMSC-derived exosomes

Exosome isolation was carried out following a previously published protocol [25]. Briefly, hucMSCs were cultured and reached 50–60% confluence. They were then placed in a serum-free MSC medium (Nuwacell Biotechnology, RP02010-1 and RP02010-2, China). After 48 h of cultivation, the medium was obtained and centrifuged for 10 min at 300*g* and for 30 min at 10,000*g*. Next, the supernatant was filtered with a 0.22-μm filter (Millipore Corp, USA) and further ultracentrifuged at 100,000 *g* for 70 min in an SW32Ti rotor (Beckman Coulter, USA). After removing the supernatant, the pellet was resuspended in PBS and then centrifuged at 100,000*g* for 70 min again. Finally, the pellet was stored at − 80 °C after being collected in 200–300 μL of PBS.

The particle size distribution of hucMSC-Exo was analyzed using a nanoparticle tracking analysis system (ZetaView, Germany). The characteristic morphological observation of the exosomes was performed with a transmission electron microscope (FEI Tecnai Spirit G2). Western blot analysis was performed to identify the expression of exosome-specific markers TSG101 (ab133586, Abcam, 1:1000 dilution), CD9 (A1703, Abclone, 1:1000 dilution), CD63 (25682-1-AP, Proteintech, 1:1000 dilution), and heat shock protein HSP70 (A12948, Abclone, 1:1000 dilution).

### Exosome labeling

Purified exosomes were dyed with PKH67 (a green fluorescent dye; Sigma–Aldrich, Germany) following the manufacturer’s protocol. Briefly, exosomes were stained with 4 μL of PKH67 in 200 μL of Diluent C fluid for 5 min. Next, isovolumetric 1% BSA was added to stop the staining. The exosomes were washed with PBS and re-purified via ultracentrifugation. Then, PKH67-labeled exosomes were incubated with ARPE19 cells for 12 h. Fluorescence microscopy (Leica Microsystems) was used to detect the green signals in cells.

### Wound-healing assay

A total of 2 × 10^5^ cells/well were plated and serum-starved overnight. Then, scratches on ARPE-19 cell monolayers were made with a sterilized 200-μL pipette tip. Images were recorded after 0, 24, and 48 h, and the wound recovery was analyzed using ImageJ software. The migration capacity was determined using a percentage of wound closure.

### Transwell assay

ARPE-19 cells (3 × 10^4^) resuspended in 200 μL of serum-free medium were placed in the upper chamber with a polycarbonate membrane (8-μm pore size, Corning, USA). Then, 600 μL of DMEM/F12 supplemented with 10% FBS was added to the lower chamber. After incubation for 12 h, the cells were stained with 0.1% crystal violet. For visualization, the images of cultured cells were collected and counted in five different fields.

### Immunofluorescence staining

Briefly, the cells after treatment were incubated with 1% BSA and 0.2% Triton X-100 for 2 h. Then, the cells were co-cultivated with primary antibodies, namely, anti-alpha smooth muscle actin (anti-α-SMA) (#ab5694, Abcam, 1:100 dilution), anti-zonula occludens-1 (anti-ZO-1) (#61-7300, Invitrogen, 1:50 dilution), and anti-Vimentin (#ARG66302, Arigo, 1:200 dilution) antibodies at 4 °C. After that, the cells were incubated with the Cy3-conjugated donkey anti-rabbit IgG and the FITC-conjugated goat anti-mouse (Jackson ImmunoResearch Labs, USA) for 1 h. The nuclei were counterstained with DAPI (Beyotime, Shanghai), and the images were taken sequentially with a fluorescent microscope.

### Western blot analysis

RIPA (Beyotime, China) supplemented with SDS buffer and protease inhibitors (Thermo Scientific) were used to extract total proteins. The proteins were transferred onto a PVDF membrane (Millipore, MA, USA) and then blocked with 5% skimmed milk. Primary antibodies against Vimentin (ARG66302, Arigo), α-SMA (ab5694, Abcam), occludin (SAB4200593, Sigma–Aldrich), and N-cadherin (13,116, CST) were incubated with the membrane at 4 °C overnight, all of them diluted at 1:1000. Next, the cells were incubated with a corresponding secondary antibody (HRP-conjugated goat anti-rabbit antibodies, 1:5000, ab15007).

### Cells transfection with miRNA

Lipofectamine 2000 (Invitrogen) was used for transfection. The cells were cultivated in six-well plates upon cell fusion of 60%. The instructions from GenePharma (Shanghai) were followed for agomir and antagomir (100 pmol) transfection. The cells were harvested for the following efficiency and function assay 48 h after transfection.

### RNA isolation and quantitation

Total RNA was extracted from hucMSC-Exo using TRIzol Reagent (TaKaRa, Japan). Before isopropanol precipitation, the Dr. GenTLE Precipitation Carrier (#9094, TaKaRa, Japan) was added as a co-precipitant to increase the yield of exosomal RNA, which was reverse transcribed into cDNA with an Mir-X miRNA First-Strand Synthesis Kit (Cat. No. 638313, TaKaRa). qRT-PCR was performed using TB Green Premix Ex Taq II (Tli RNaseH Plus) (RR820A, TaKaRa) and then detected with an ABI 7500 qPCR instrument (Thermo Fisher Scientific). The relative expression of miRNAs or mRNAs was analyzed using the 2^−ΔΔCt^ method. U6 or GAPDH was deemed as an internal control. Primer sequences were acquired from GenePharma (Shanghai) (Table 1).

**Table 1:** Primers sequences for qRT-PCR

Primers	Sequences (5′ to 3′)
has-miR-100-5p	F′ AACCCGTAGATCCGAACTTGTG
has-miR-21-5p	F′ GCAGTAGCTTATCAGACTGATG
has-miR-27b-3p	F′ TTCACAGTGGCTAAGTTCTGC
has-miR-145-5p	F′ GGTCCAGTTTTCCCAGGA
has-miR-23b-3p	F′ AGATCACATTGCCAGGGA
has-miR-221-3p	F′ CAGAGCTACATTGTCTGCTG
has-miR-204-5p	F′ CAGTTCCCTTTGTCATCCTATG
has-miR-211-5p	F′ GCAGTTCCCTTTGTCATCCT

The 3′ primer for above Forward qPCR is the mRQ 3′ Primer, along with U6 Forward Primer and U6 Reverse Primer are supplied in Mir-X miRNA First-Strand Synthesis Kit (Cat. No. 638313)

### Dual-luciferase reporter assay

The cDNA of HOXC6 was loaded onto a psiCHECK2 vector (Promega, USA) (HOXC6- wild). Mutations were induced in the potential miR-27b-3p-binding sites using a Fast Mutagenesis kit V2 (Vazyme, China) (HOXC6-mut). The luciferase activity was detected using a Dual-Luciferase Reporter Assay System (Promega).

### Laser-induced CNV model and drug administration

C57BL/6 J mice aged 6–8 weeks were used in this study. They were housed at the Laboratory Animal Center of Shanghai General Hospital. The laser-induced subretinal fibrosis model was established as previously described and observed for 35 days to generate subretinal fibrosis [1]. In brief, four to six laser spots (532 nm, 180 mW, 100 ms; Novus Spectra, Japan) were selected at each fundus around the optic disc. The disruption of Bruch’s membrane was confirmed by subretinal bubble formation immediately after laser application. HucMSC-Exo or 2 μL of PBS was injected into the vitreous cavity immediately after laser injury. After injection, the mice were randomly sacrificed (*n* = 15) on days 7, 21, and 35 for further quantification of CNV and subretinal fibrosis.

### Choroidal flat-mount and immunofluorescence staining

The areas of CNV and collagen fibers were determined on choroidal flat mounts on days 7, 21, and 35. Mouse eyecups were fixed in 4% paraformaldehyde (PFA), and anterior segments were removed before cutting four to six radial incisions to be flattened. Then, the RPE–choroid complexes were washed, blocked with 5% goat serum albumin and 0.3% Triton X-100, and then incubated with FITC-labeled isolectin-B4 (IB4) (Vector Laboratories, 1:100) (for evaluating CNV) and collagen type I antibody (ab34710, Abcam, dilution 1:100) (for evaluating subretinal fibrosis) at 4 °C overnight. The secondary antibody against collagen type I was Cy3-conjugated donkey anti-rabbit IgG (Jackson ImmunoResearch Labs, 1:200).

### Hematoxylin and eosin and Masson staining

The eyes were removed after 7, 21, and 35 days; fixed for 2 h at 4 °C in 4% PFA; and then dehydrated and embedded in paraffin. Hematoxylin and eosin staining was conducted following specific protocols. Masson’s trichrome staining was performed with a trichrome staining kit (ab150686, Abcam). The rate was automatically averaged using ImageJ (MD, USA).

### Statistical analysis

GraphPad Prism Version 8.2 was used for statistical analysis. All data were expressed as mean ± standard deviation (SD). The statistical analysis between two sets of data was performed with the Student *t* test. Comparisons between multiple groups were analyzed by one-way analysis of variance. A *P* value less than 0.05 indicated a statistically significant difference.

## Results

### Isolation and identification of hucMSCs

**Fig. 1:**
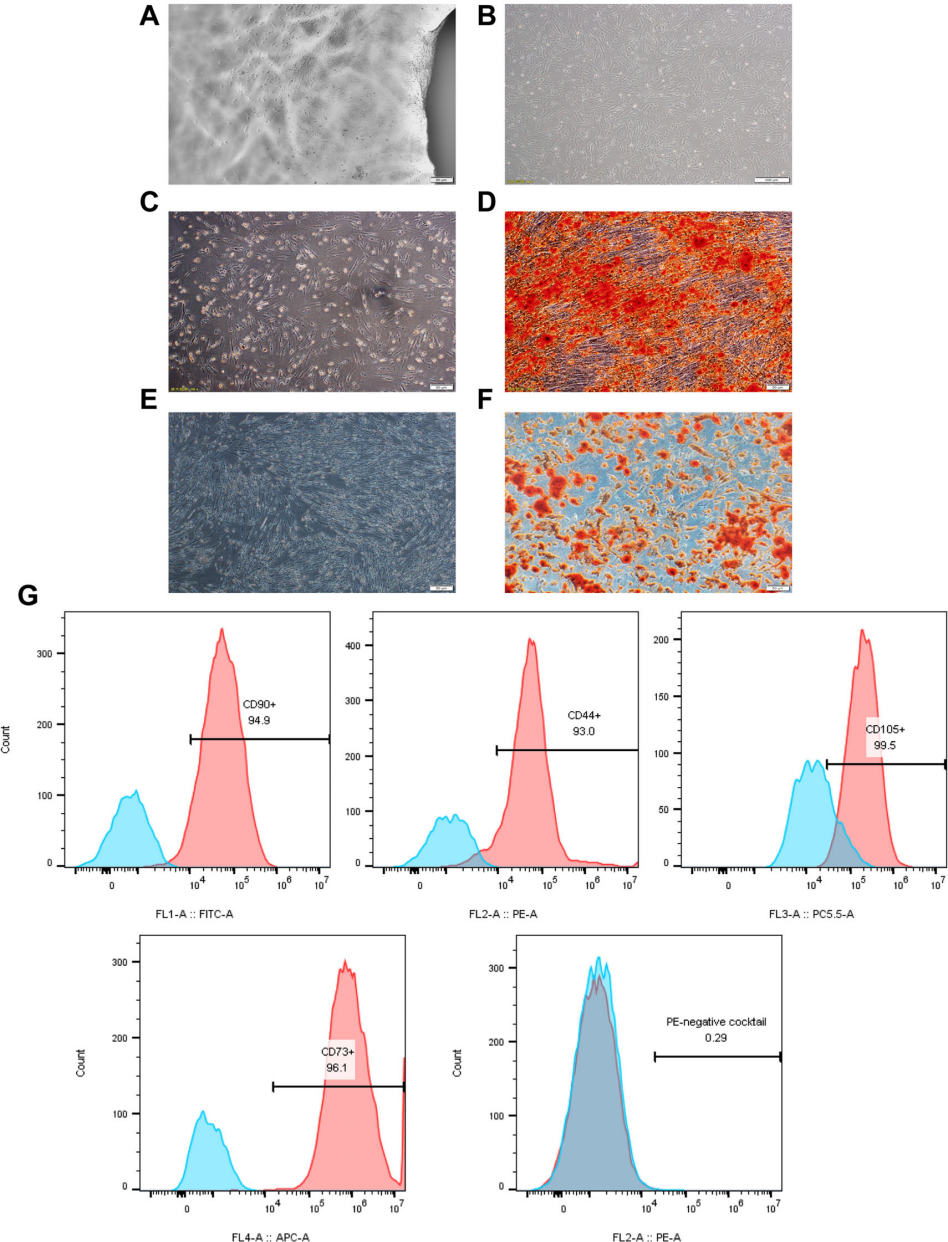
Characteristics of hucMSCs. **a**, **b** Cell morphology of passage 0 (bar = 50 μm) and passage 3 (bar = 200 μm) human umbilical cord mesenchymal stem cells (hucMSCs) observed under an inverted microscope (bar = 50 μm). **c**, **d** Osteogenic differentiation of hucMSCs was performed in the differentiation medium, while the control cells were grown in a regular medium. Both of them were dyed with Alizarin Red S staining. Most hucMSCs were Alizarin red positive. **e**, **f** Oil red O staining was used to stain hucMSCs after adipogenic differentiation, showing the Oil red O-positive lipid droplets. **g** Flow cytometry analysis of the phenotypic markers of hucMSCs showed that hucMSCs were positive for CD90, CD44, CD105, CD73, and CD105 and negative for CD34, CD45, CD11b, CD19, and human leukocyte antigen (HLA)-DR

HucMSCs were successfully isolated from Wharton’s jelly region of umbilical cords. After culturing for 2 weeks, the cells around tissue blocks displayed a fibroblast-like morphology and attached to the plastic surface (Fig. 1a). After passage, they grew rapidly and arranged in a spiral pattern (Fig. 1b). MSCs could undergo differentiation into osteocytes and adipocytes, and their multipotency was detected using Oil red O staining (Fig. 1c and d) and Alizarin red, respectively (Fig. 1e and f). The expression of surface markers of hucMSCs was determined by flow cytometry. The positive markers of CD73, CD90, and CD105 along with negative cocktail markers (CD19, CD34, CD45, CD14, and HLA-DR) are shown in Fig. 1g. A mouse isotypic IgG was used as a control. These results showed that hucMSCs were successfully isolated and identified.

### Exosome purification, characterization, and internalization into cells

**Fig. 2:**
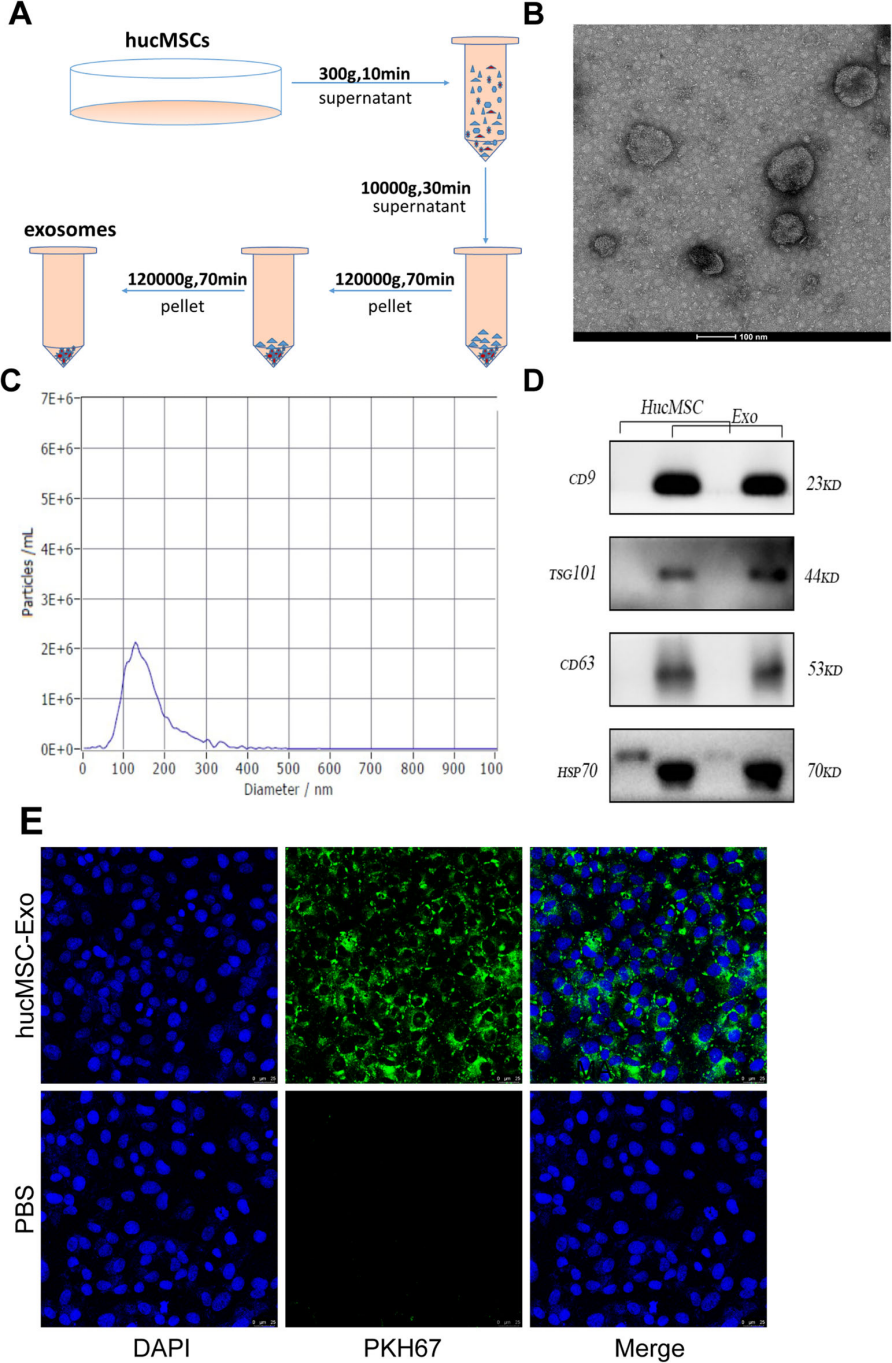
Identification of hucMSC-derived exosomes (hucMSC-Exo). **a** The hucMSC-Exo were isolated from the conditioned medium using serial centrifugation. **b** Transmission electron microscopic images of typical hucMSC-Exo. Scale bar = 100 nm. **c** Size distribution of the hucMSC-Exo was determined using a nanoparticle tracking analysis. hucMSC-Exo had an original concentration of 8.8e+9 particles/mL, a mean size of 159.0 nm, and a peak size of 128.6 nm. **d** Positive expression of markers CD9, TSG101, CD63, and HSP70 was mainly detected in hucMSC-Exo using Western blot analysis. **e** Fluorescence result of ARPE19 cells after being co-cultured with hucMSC-Exo labeled with PKH67, observed under a confocal microscope (scale bar = 25 μm)

HucMSC-Exo were purified from the third- to seventh-generation medium by serial centrifugation as described in Fig. 2a. As shown in Fig. 2b, the round or oval membranous vesicle morphology of exosomes was evaluated under a transmission electron microscope. The nanoparticle tracking analysis showed that the distribution mainly focused on 30- to 160-nm particle size (Fig. 2c). Western blot analysis showed that traditional exosomal markers (CD9, TSG101, CD63, and HSP70) were expressed obviously in isolated hucMSC-Exo (Fig. 2d). Therefore, it was confirmed that hucMSC-Exo were isolated successfully. Furthermore, PKH67-labeled exosomes were observed under a confocal microscope. The result revealed that exosomes were internalized by ARPE19 cells and intensively distributed around the nucleus (Fig. 2e).

### hucMSC-Exo alleviated laser-induced CNV and subretinal fibrosis in mice

**Fig. 3:**
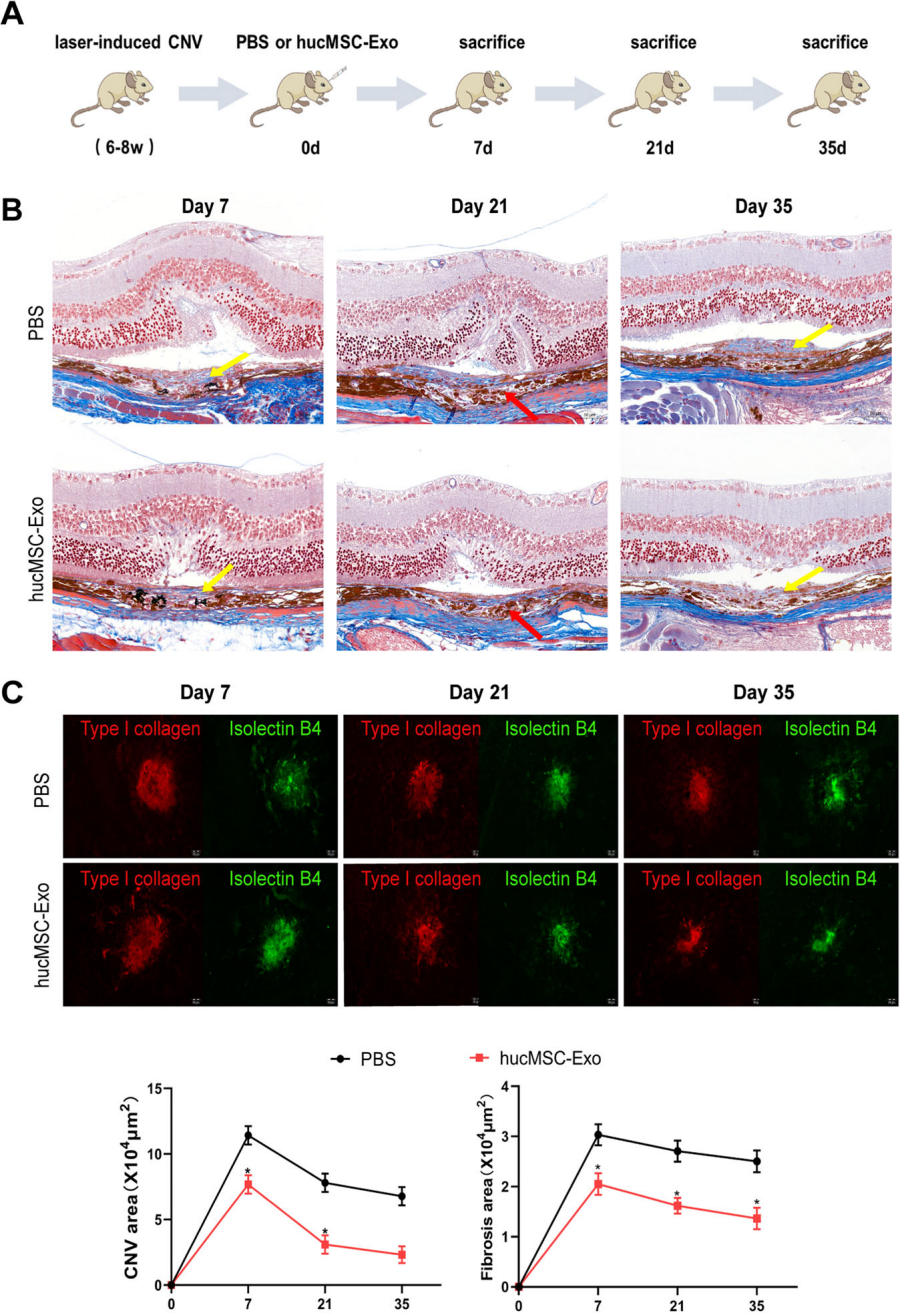
hucMSC-Exo treatment had less fibrosis development in vivo. **a** Study design. **b** The RPE–Bruch membrane complex was disrupted, the normal arrangement of the inner nuclear layer was lost and neovascular hide beneath the subretinal space 7 days after the laser burn. Compared with the PBS group, the number of collagen fibers reduced (blue) in the group treated with hucMSC-derived exosomes 35 days after laser photocoagulation. **c** Choroidal neovascularization (CNV; dyed with isolectin B4) and fibrosis (dyed with collagen type I) were recorded 7, 21, and 35 days after laser burn in the PBS and hucMSC-Exo groups. **d** Mean volume of the CNV and fibrosis 7, 21, and 35 days after laser burn

Laser-induced CNV and subretinal fibrosis model were established and intravitreally injected with hucMSC-Exo immediately to explore their influence on subretinal fibrosis in vivo (Fig. 3a). Masson staining performed on choroidal flat mounts revealed that an obvious disruption change was observed in the choroidal layer and outer nuclear layers within the center of laser burn 7 days after laser photocoagulation. Subsequently, newly formed vessels and retinal edema were found to be extended into the subretinal space 7 days and 21 days, respectively, after laser photocoagulation. The collagen area obviously decreased in the hucMSC-Exo group compared with the control group after 21 and 35 days (Fig. 3b, *P* < 0.05). Furthermore, the immunofluorescence staining of choroidal flat-mount indicated that the area of CNV was the largest on day 7 and declined gradually between day 21 and day 35. Importantly, fewer vascular channels (isolectin-B4) and area of fibrosis (type 1 collagen) were observed in the subretinal space after 21 days in the hucMSC-Exo group (Fig. 3c, *P* < 0.05). These findings indicated that hucMSC-Exo strongly reduced laser-induced subretinal fibrosis in mice.

### hucMSC-Exo inhibited cell migration

**Fig. 4:**
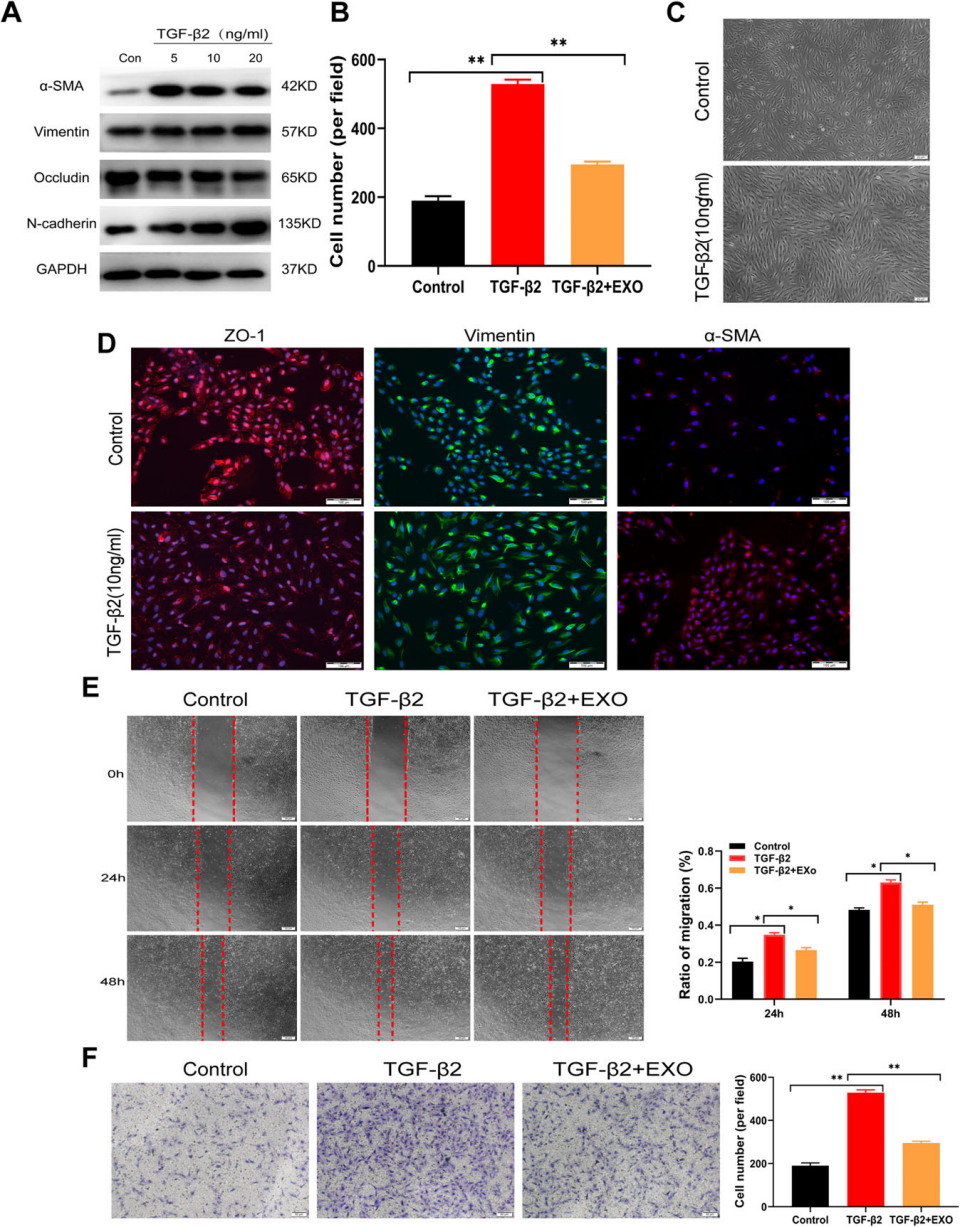
Effects of hucMSC-Exo on cell migration. **a**, **b** Effect of different concentrations of hucMSC-Exo on the expression of occludin, Vimentin, N-cadherin, and α-SMA in TGF-β2-treated ARPE19 cells. **c** Typical morphological alteration of ARPE19 cells in the untreated group or in the 10 ng/mL TGF-β2-treated group. **d** Immunocytochemistry of ARPE19 cells. The cells were fixed with PFA and dyed with primary antibodies against ZO-1, α-SMA, and Vimentin. Scale bar = 20 μm. **e** Images of cells were recorded 0, 24, and 48 h after a scratch. Scale bar = 50 μm. **f** Harvested cells were plated on the upper chamber and migrated for an additional 12 h. The migrated cells were recorded under a phase-contrast microscope. Statistics are presented as means ± SD. *P < 0.05, **P < 0.01, ***P < 0.001

TGF-β2-induced EMT has been investigated in numerous cell lines as fibrosis models, and ARPE19 cells are one of the leading effector cells in subretinal fibrosis [26, 27]. Hence, different concentrations of TGF-β2 were added to ARPE19 cell media to establish a successful RPE EMT model and further to verify the roles of hucMSC-Exo in the subretinal fibrosis environment. When ARPE-19 cells were treated with diverse concentrations of TGF-β2 for 48 h, the expression of epithelial markers was downregulated, whereas the expression of mesenchymal markers, such as N-cadherin, Vimentin, and α-SMA, was upregulated at the protein level (Fig. 4a and b). The stimulation of ARPE-19 cells with 10 ng/mL TGF-β2 resulted in a significant morphological alteration, with a transition from typical cobblestone-like cells to elongated spindle-shaped mesenchymal-like cells (Fig. 4c). The stimulated cells displayed a loss of epithelial characteristics and the acquisition of mesenchymal characteristics under the immunofluorescence microscope compared with the control group, (Fig. 4d). These results suggested that ARPE-19 cells underwent a process of EMT after treatment with 10 ng/mL TGF-β2.

Additionally, the scratch assays showed that the cells treated with TGF-β2 exhibited an enhanced migratory ability, while the cells treated with MSC-derived exosomes exerted an opposite effect after 24 and 48 h (*P* < 0.05) (Fig. 4e). Similar results were observed in the Transwell assay, indicating that fewer cells migrated through the membrane in the hucMSC-Exo group than in the TGF-β2-treated group after 12 h of incubation (Fig. 4f). These assays consistently demonstrated that exosomes derived from MSC treatment significantly inhibited cell migration.

### hucMSC-Exo inhibited the expression of EMT-associated proteins

**Fig. 5:**
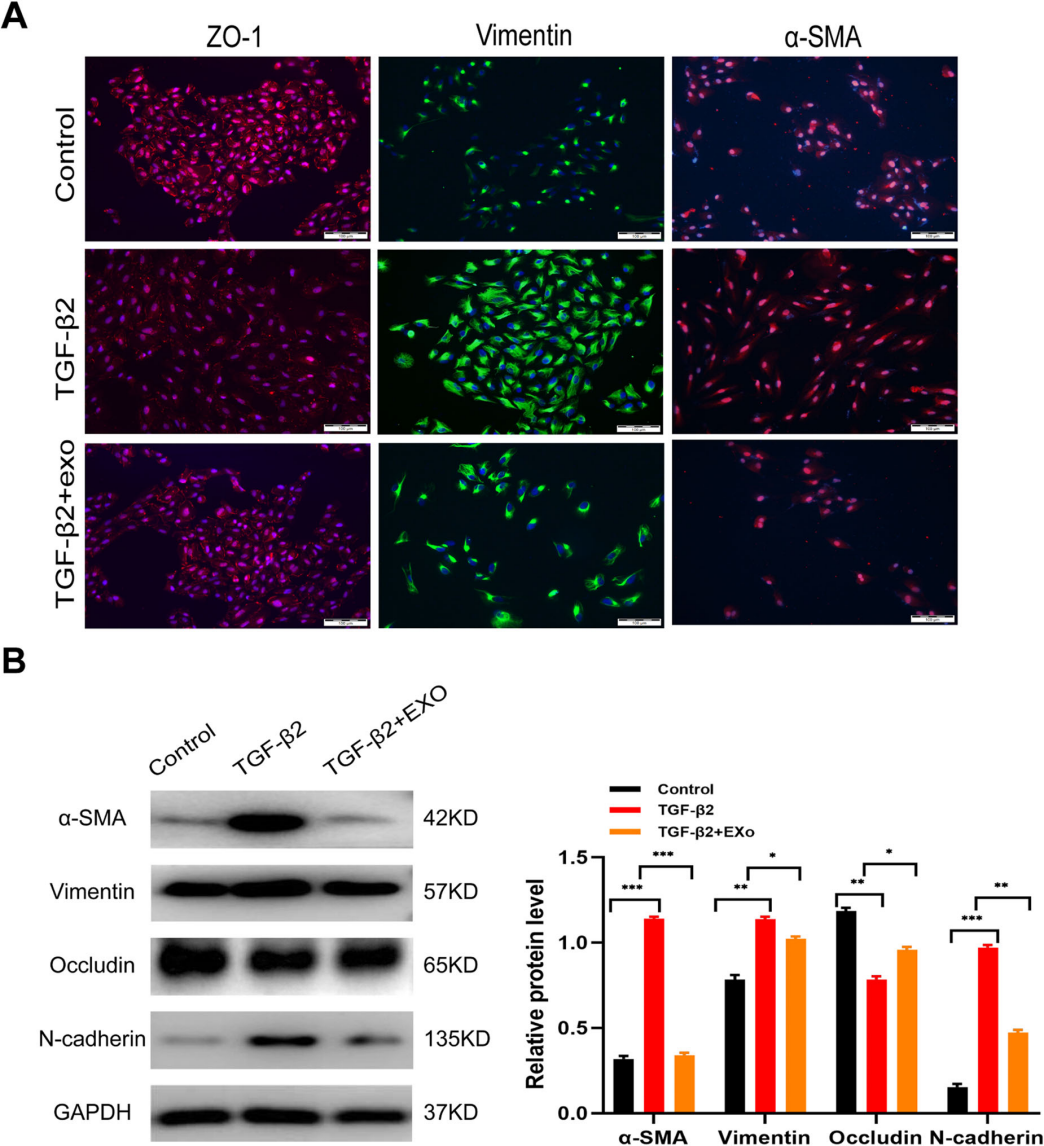
Inhibitory effect of hucMSC-Exo on TGF-β2-treated EMT in ARPE19 cells. **a** Immunofluorescence staining of Vimentin, ZO-1, and a-SMA expression in ARPE19 cells treated with or without 100 μg/mL hucMSC-Exo; the nuclei were stained with DAPI. **b** Extracted total protein was loaded and analyzed by Western blot analysis. The relative protein expression was quantified. The data represent the average of three independent experiments. Statistics are presented as means ± SD. *P < 0.05, **P < 0.01, ***P < 0.001

The alteration of representative proteins is an important feature of RPE cells in the subretinal fibrosis environment. The expression of EMT-related indicators and cell adhesion markers was examined to evaluate the effect of hucMSC-Exo in ARPE19 cells. Immunofluorescence results revealed that the expression of intercellular tight junction ZO-1 was abrogated by TGF-β2, which was alleviated by hucMSC-Exo. The mesenchymal proteins α-SMA and Vimentin were distributed around the cells because of TGF-β2 induction. However, their expression markedly decreased on exposure to hucMSC-Exo in combination with TGFβ2 compared with the TGF-β2-alone group (Fig. 5a). Besides, hucMSC-Exo significantly upregulated the expression of occludin while decreasing the expression of mesenchymal markers (Vimentin, N-cadherin, and α-SMA) compared with the TGF-β2 group (Fig. 5b). The aforementioned results indicated that hucMSC-Exo reduced subretinal fibrosis through suppressing EMT.

### Detection of miR-27b expression in hucMSC-Exo

**Fig. 6:**
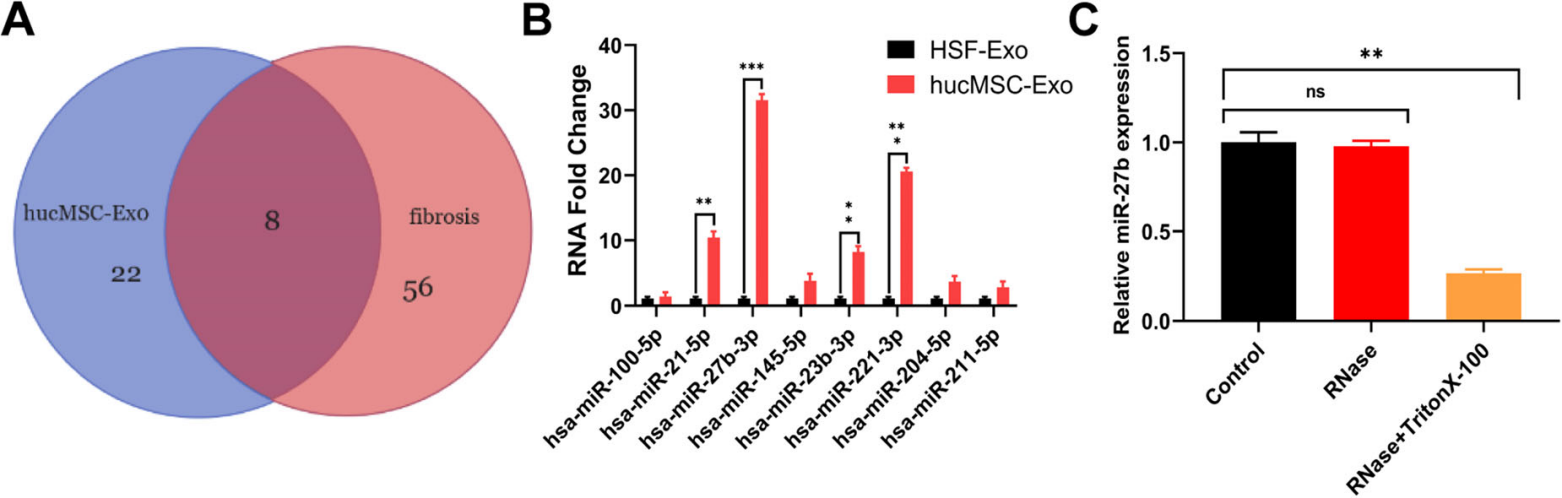
Relative miR-27b-3p expression in exosomes. **a** Candidate miRNA were screened using the Venn diagram package. **b** Predictions of eight potential targeted miRNAs of hucMSC-Exo were detected by qRT-PCR; HSF-Exo was used as the reference. Different-color areas represent different datasets. **c** Average miR-27b levels in hucMSC-Exo after treatments with RNase and Triton X-100 for 30 min, relative to miR-27b levels in the control untreated group, normalized to U6. Statistics are presented as means ± SD. *P < 0.05, **P < 0.01, ***P < 0.001

Studies have shown that miRNAs are enriched in MSC-derived exosomes and play a pivotal role in cellular communication. The present study aimed to identify the miRNAs that contributed to the therapeutic effects of hucMSC-Exo on subretinal fibrosis. Eight miRNAs were strongly enriched in hucMSC-Exo and closely correlated with fibrosis (Fig. 6a). Next, the expression of these miRNAs was detected using qRT-PCR. The results indicated that miR-27b-3p expression was the highest in hucMSC-Exo relative to HSF-Exo (Fig. 6b). The average miR-27b levels relative to U6 in hucMSC-Exo after treatment with RNase (0.5 μg/μL) and 0.1% Triton X-100 for 30 min were measured by qRT-PCR, which verified the presence of miR-27b in hucMSC-Exo. As shown in Fig. 6c, the exosomes treated with RNase did not show a significantly low level of miR-27b unless they were co-treated with Triton X-100, which degraded the exosomal membrane (Fig. 6c). Hence, miR-27b was protected within the intact exosomes from RNase degradation, which was considered to be a real EV-miRNA. Furthermore, the elevated levels of miR-27 were confirmed in hucMSC-Exo. Therefore, miR-27b-3p was selected as a potential candidate to mediate the therapeutic effects of hucMSC-Exo in the model.

### hucMSC-Exo transferred a high level of miR-27b to ARPE19 cells

**Fig. 7:**
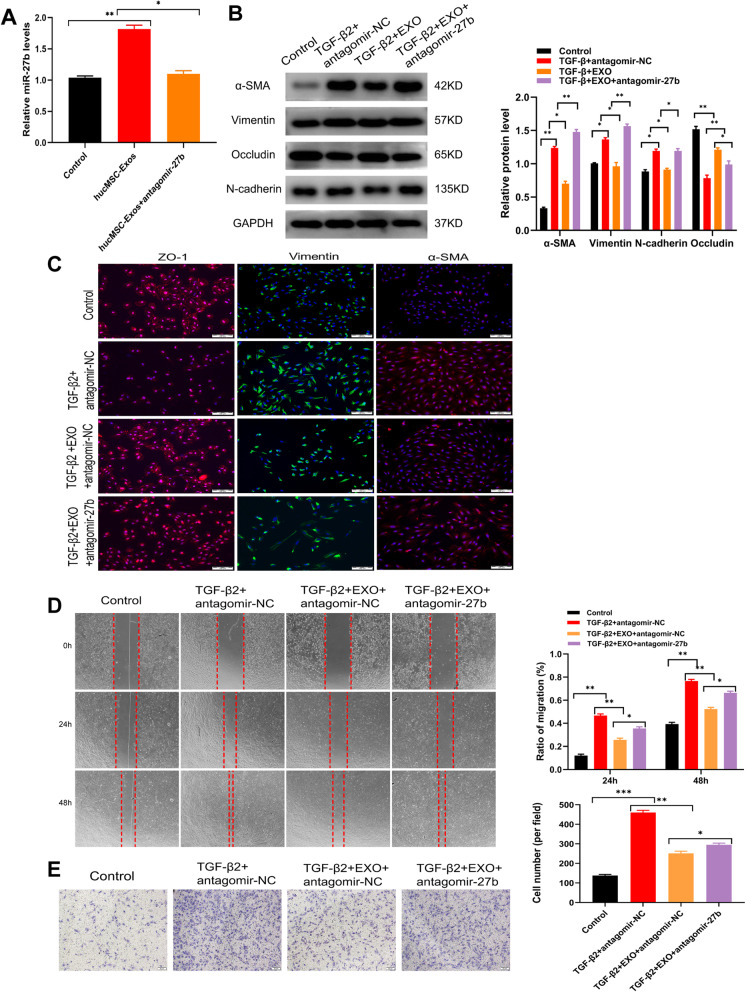
hucMSC-Exo were enriched with miR-27b-3p that could suppress the EMT process. **a** qRT-PCR showed that the application of antagomiR-27b-3p could counteract the overexpression of miR-27b-3p caused by hucMSC-Exo. **b** Western blot analysis indicated that antagomiR-27b-3p could recover the EMT process suppressed by hucMSC-Exo. **c** Effect of antagomiR-27b-3p on the EMT-related protein changes assessed by immunofluorescence. **d–e** Effects of antagomiR-27b-3p on the migration and wound-healing ability of ARPE19 cells were measured by Transwell and scratch assays; hucMSC-Exo was used as a reference. Statistics are presented as means ± SD. *P < 0.05, **P < 0.01, ***P < 0.001

ARPE19 cells treated with exosomes were transfected with an miR-27b-3p inhibitor (antagomiR-27b-3p) to explore the effect of miR-27b-3p in hucMSC-Exo on TGFβ2-induced ARPE-19 cells. The results showed a marked decline in miR-27b-3p expression following antagomiR-27b-3p transfection (Fig. 7a). Therefore, the study confirmed that transferring miR-27b-3p was one of the mechanisms for hucMSC-Exo to suppress TGF-β2-meditated EMT in ARPE19 cells. The transfection with antagomiR-27b-3p led to the downregulation of an epithelial marker occludin, while increasing the expression of mesenchymal markers (Vimentin, N-cadherin, and α-SMA) compared with the hucMSC-Exo group (Fig. 7b). Consistently, scratch and Transwell assays also confirmed that the migration of cells treated with hucMSC-Exo was enhanced if they were also transfected with antagomiR-27b-3p (Fig. 7d and e). The study similarly confirmed that hucMSC-Exo enhanced the expression of tight junction ZO-1 destroyed by TGF-β2, and this effect was eliminated by additional antagomiR-27b-3p treatment. In addition, the expression of mesenchymal proteins α-SMA and Vimentin significantly increased on exposure to antagomiR-27b-3p compared with the control group, as detected by immunofluorescence (Fig. 7c). Together, these results revealed that hucMSC-Exo was enriched in miR-27b-3p, which could regulate the functionality of TGFβ2-induced ARPE-19 cells.

### Enhancing the expression of miR-27b inhibited TGFβ2-induced EMT

**Fig. 8:**
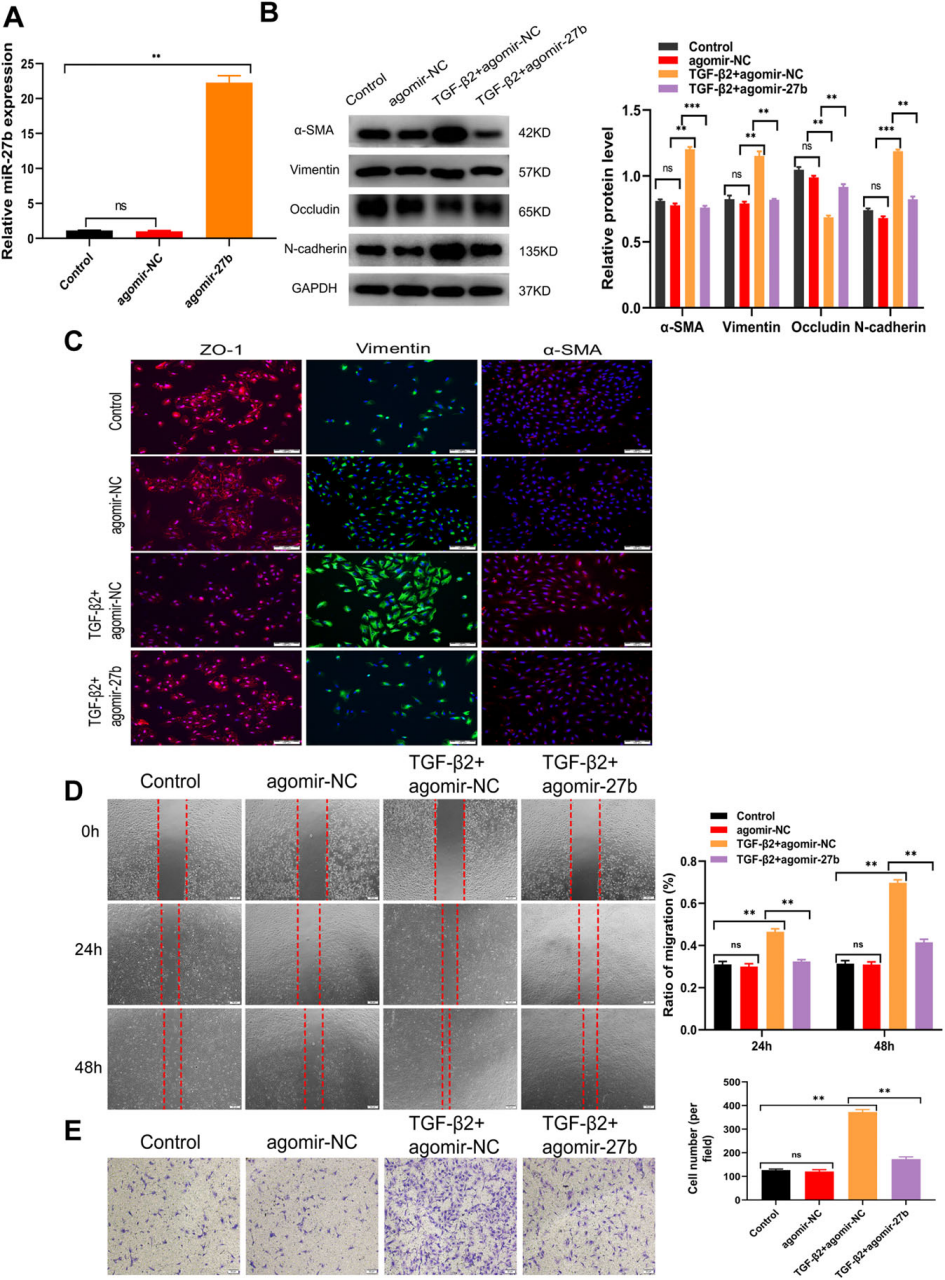
miR-27b-3p inhibited cell migration and expression of EMT-associated proteins in vitro. **a** miR-27b-3p level in three groups analyzed by qRT-PCR. **b** Effect of miR-27b-3p on the expression of EMT-related proteins occludin, Vimentin, N-cadherin, and α-SMA evaluated by Western blot analysis. **c** Immunofluorescence was further used to assess cell morphological alteration. **d–e** Effects of miR-27b-3p on the migration and wound-healing ability of ARPE19 cells. Statistics are presented as means ± SD. *P < 0.05, **P < 0.01, ***P < 0.001

A series of experiments were conducted to gain insights into whether miR-27b-3p could exert an impact on TGF-β2-induced EMT. First, the transfection efficiency of agomiR-27b-3p was detected by qRT-PCR (Fig. 8a). Also, the addition of miR-27b-3p markedly rescued the loss of epithelial phenotype ZO-1 and inhibited the EMT of ARPE19 cells compared with the TGF-β2 group, as demonstrated by immunofluorescence and Western blot analysis (Fig. 8b and c). Furthermore, the activation of TGF-β2-induced migration was obviously suppressed by miR-27b-3p overexpression compared with that in RPE cells without miR-27b-3p pretreatment (Fig. 8d and e). Together, the results confirmed that miR-27b-3p could directly inhibit the EMT of TGFβ2-induced ARPE19 cells in vitro.

### Identification of candidate genes regulated by miR-27b

**Fig. 9:**
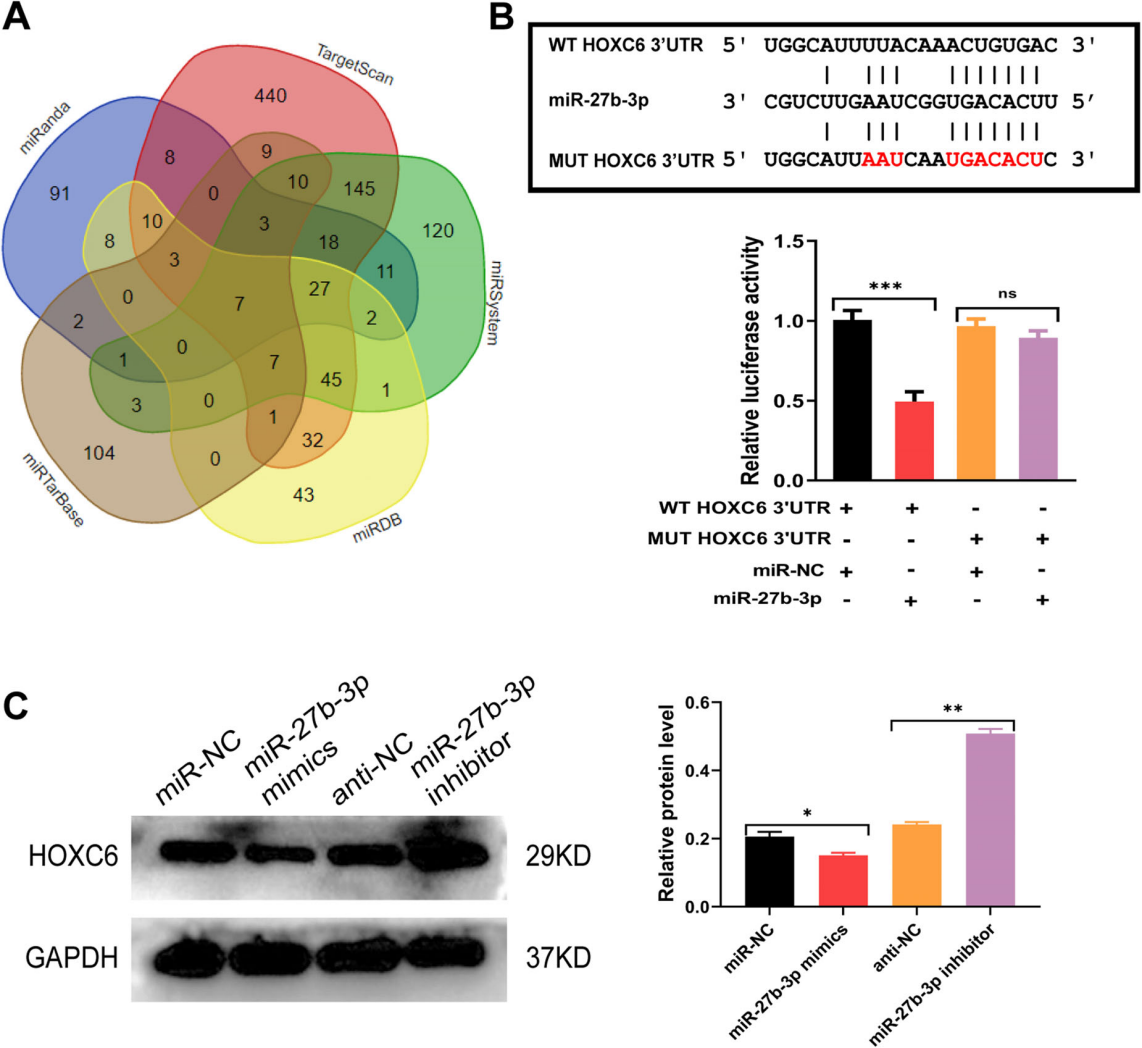
miR-27b-3p modulated the EMT process by suppressing HOXC6. **a** Potential targets of miR-27b-3p were predicted using five databases (TargetScan, miRanda, miRtarbase, miRSystem, and miRDB). **b** The targeted relationship of miR-27b-3p with HOXC6 was verified by the dualluciferase assay. **c** Effect of miR-27b-3p on the expression of HOXC6 assessed by Western blot analysis. Data are the means ± SD of three independent experiments. *P < 0.05, **P < 0.01, ***P < 0.001

As miR-27b markedly suppressed EMT in vitro, the present study concentrated on explicating the potential target of miR-27b in RPE cells. Bioinformatics analysis was performed with five online analysis instruments (miRanda, miRTarbase, TargetScan, miRDB, and miRsystem). As a result, seven genes were predicted to be direct targets of miR-27b-3p, including HOXC6 (Fig. 9a). Previous studies reported that HOXC6 played a vital role in EMT [28–30]; therefore, it was chosen as the candidate. In addition, the dual-luciferase reporter assay was applied to confirm whether HOXC6 was a target for miR-27b-3p. The results illustrated that miR-27b-3p overexpression inhibited the luciferase activity of psiCHECK-2-HOXC6-WT, but it did not affect the luciferase activity of psiCHECK-2-HOXC6-MT (Fig. 9b). These data indicated that HOXC6 was a binding target of miR-27b-3p. Consistently, Western blot analysis indicated that the expression of HOXC6 was significantly downregulated in ARPE19 cells following transfection with miR-27b-3p mimics but exerted the opposite effect in ARPE19 cells transfected with the miR-27b-3p inhibitor (Fig. 9c), indicating that HOXC6 was a direct target of miR-27b-3p.

## Discussion

Subretinal fibrosis defines the end stage of nAMD, leading to irreversible vision loss. Currently, no treatment is available to prevent or treat nAMD [1]. Thus, novel therapeutic methods need to be urgently developed to prevent vision loss and reduce subretinal scar formation in nAMD. The intravitreal administration of MSC has been reported to have an encouraging therapeutic effect on multiple retinal disease models, including retinal damage, inflammation, and nerve injury [23]. Despite the beneficial effects of MSCs in repairing retinal injury and restoring the visual system, their application is confined to ethical issues, poor diffusion through biological barriers, and possible complications such as epiretinal membranes, inflammatory response, and malignant transformation [31]. It is now clear that they exert most of their biological effects through the paracrine secretion of soluble factors and exosomes [21]. MSC-derived exosomes have been shown to own all the superiority of MSCs in limiting the extent of damage, reducing apoptosis, and restricting inflammation [16, 20, 32]. Importantly, exosomes have a reliable stability, a better ability to penetrate deep tissues and avoid immune attack, a lower chance of immunological rejection, and no risk of malignant transformation or vitreoretinal proliferation and uncertain differentiation in MSCs [33, 34].

MicroRNA, a class of small noncoding RNAs, serves as a regulator of gene expression at the post-transcriptional level, causing a translational arrest or mRNA degradation [35]. They are involved in regulating basic cellular processes such as proliferation and differentiation [36]. In addition, many studies analyzed the miRNA disorders in patients with AMD, suggesting that miRNA-based treatment might serve as a promising novel strategy to ameliorate AMD-related tissue damage [37]. The preliminary literature screening and PCR results indicated that miR-27b-3p from hucMSC-Exo might be one of the critical miRNAs in subretinal fibrosis. MiR-27b was discovered to be observably upregulated in nAMD plasma, and the upregulation of miR-27b markedly blocked the cell proliferation and migration in PDGF-BB-induced ARPE19 cells [38, 39]. Various growth factors, including platelet-derived growth factor (PDGF), hepatocyte growth factor (HGF), connective tissue growth factor (CTGF), and transforming growth factor-β (TGF-β), are involved in the pathogenesis. However, the present study focused on TGF-β2 because it was predominant in aqueous humor and its concentration in the vitreous humor correlated with the progression of retinal fibrosis. Moreover, PDGF, TGF-β1, and CTGF are known to be targets of TGF-β2 signaling, implying that TGF-β2 could mediate the secondary effects of these other factors on EMT and fibrosis [40]. Importantly, previous studies demonstrated that the suppression of TGF-β2-induced EMT could reduce the development of collagen fibers and fibrotic membrane and thereby protect against fibrosis-relative retinal diseases [41]. No relevant report shows the involvement of miR-27b in the EMT of RPE cells induced by TGF-β2. Therefore, the present study aimed to explore the effects of hucMSC-Exo and miR-27b on subretinal fibrosis in vivo and in vitro and further investigate its anti-fibrotic mechanism.

**Fig. 10:**
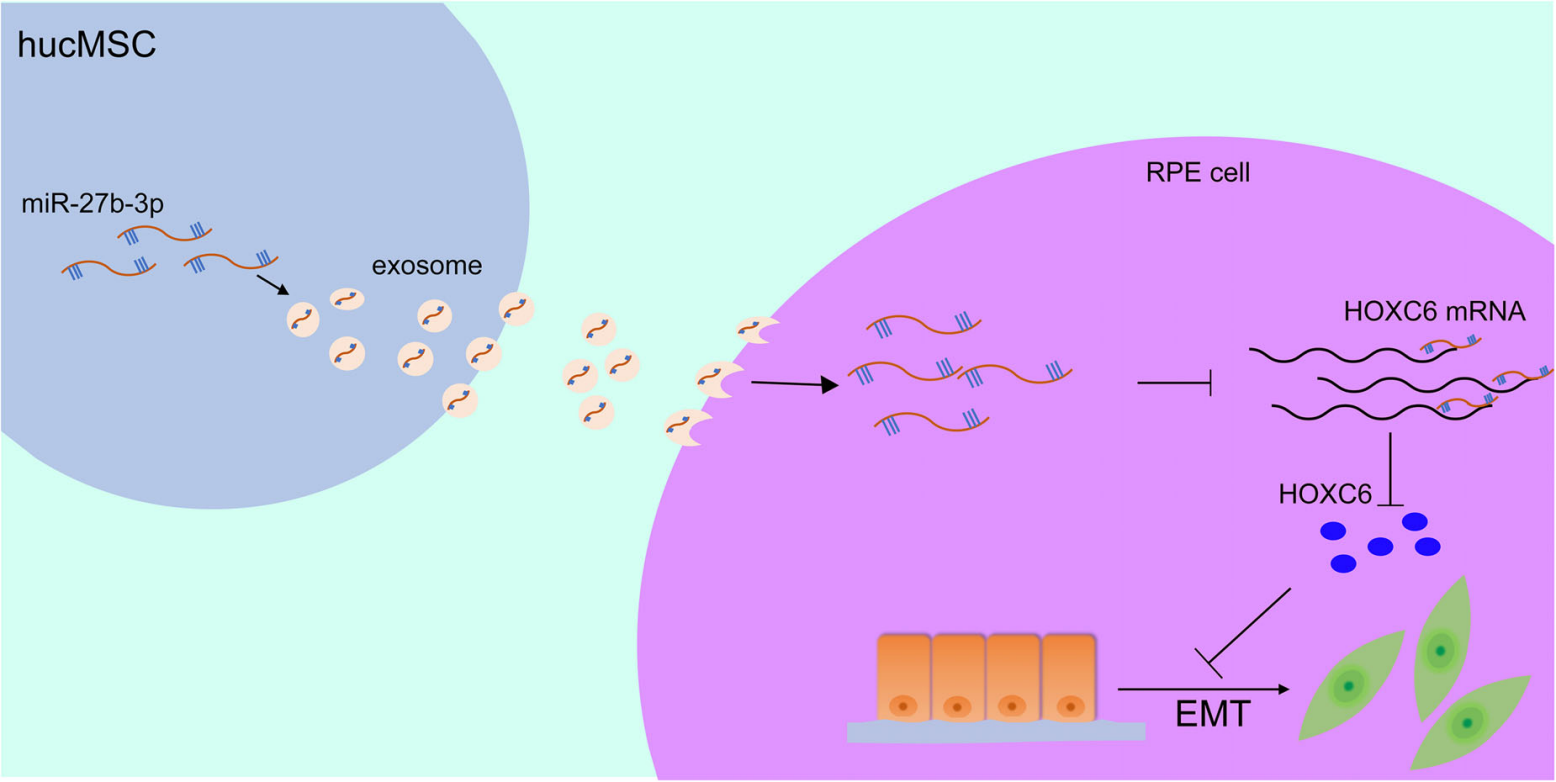
Schematic representation portrays that hucMSC-derived exosomal miR-27b-3p could alleviate subretinal fibrosis via suppressing epithelial–mesenchymal transition by targeting HOXC6

This study was novel in demonstrating that hucMSC-Exo could alleviate subretinal fibrosis, including the decrease in both CNV and subretinal fibrosis, reduction of migration, and reversal of EMT markers in TGF-β2-induced RPE cells. Moreover, hucMSC-Exo inhibited subretinal fibrosis by delivering miR-27b because the inhibitory effects were weakened when silencing the expression of miR-27b in hucMSC-Exo, but rescued after miR-27b overexpression. Bioinformatics analysis and luciferase reporter assay revealed that miR-27b-3p directly targeted the 3′-UTR of HOXC6. Consistently, the upregulation of miR-27b-3p markedly downregulated HOXC6 expression at the protein level (*P* < 0.05). HOXC6, a member of the homeobox superfamily, plays pivotal roles in numerous cellular processes, including cell apoptosis, proliferation, and differentiation [42]. Studies have shown that the silencing of HOXC6 suppressed EMT via the inhibition of the TGF-β/Smad signaling pathway in cervical carcinoma cells [28]. Therefore, miR-27b-3p is likely to block the activation of the TGF-β/Smad signaling pathway in RPE cells partly by targeting the HOXC6 gene. Taken together, these results suggest that miR-27b-3p/HOXC6 axis might be a downstream mediator of the recovery pathway induced by hucMSC-Exo (Fig. 10).

Notably, it is reported that the growth of CNV can break through Bruch’s membrane and results in subretinal fluid, retinal or subretinal hemorrhage, or subretinal fibrosis in clinical practice [43]. Previous studies demonstrated that MSC-Exo had a great potential to downregulate the expression of VEGF in RPE cells, which was increased at an early stage in the laser-induced retina injury model and seemed to reduce the leakage of the vessel in a model of AMD [19]. However, in the mouse model of laser-induced CNV and subretinal fibrosis in this study, significant reductions were observed in CNV and fibrotic scars in mice within the injection dose range mentioned in a previous study, but no signs of fundus bleeding were observed. Whether its anti-fibrosis effect is dose dependent needs to be further explored. In addition, no adverse reactions were observed during the entire follow-up process after the intravitreal injection of hucMSC-Exo.

This study had several limitations. First, miR-27b-3p is just one of the miRNAs with significantly increased levels in hucMSC-Exo compared with HSF-exosomal miRNAs. Although hucMSC-Exo strongly inhibited the activation of EMT in RPE cells and reduced subretinal fibrosis, the roles of other miRNAs also deserve further investigation. Second, the experiments strongly demonstrated that miR-27b-3p had an obvious inhibitory effect on the EMT of ARPE19 cells; however, the proposed mechanisms need to be extensively tested in vivo. Finally, a large number of studies demonstrated that miRNAs packaged in MSC-Exos were functional molecules mediating beneficial effects [44], but the therapeutic effect of proteins and other beneficial components also deserves further research. In brief, it was believed that MSC-derived exosomes had great potential to be superior candidates in the therapy of retinal diseases in various clinical stages. An in-depth study should be conducted on the effect and mechanism of action of hucMSC-Exo in the future.

## Conclusions

The hucMSC-derived miR-27-3p-enriched exosomes effectively suppressed the activation of EMT in RPE cells and alleviated subretinal fibrosis by inhibiting the process of EMT in TGFβ2-treated RPE cells. The present study provided a possible therapeutic tool for treating fibrosis-related retinal disease, as well as insights into the mechanism of hucMSC-Exo in subretinal fibrosis.

## Data Availability

The authors confirm that the data supporting the findings of this study are available within the article.

## Abbreviations

ARVO: Association for Research in Vision and Ophthalmology
α-SMA: α-Smooth muscle actin
CNV: Choroidal neovascularization
CTGF: Connective tissue growth factor
DMEM: Dulbecco’s modified Eagle medium
EMT: Epithelial–mesenchymal transition
HucMSCs-Exo: Human umbilical cord mesenchymal stem cell-derived exosomes
H&E: Hematoxylin and eosin
HGF: Hepatocyte growth factor
MSC: Mesenchymal stem cell
nAMD: Neovascular age-related macular degeneration
PFA: Paraformaldehyde
PDGF: Platelet-derived growth factor
RPE: Retinal pigmented epithelium
TEM: Transmission electron microscopy

## References

[1] Ishikawa K, Kannan R, Hinton DR (2016). Molecular mechanisms of subretinal fibrosis in age-related macular degeneration. Exp Eye Res.

[2] Wu D, Kanda A, Liu Y, Kase S, Noda K, Ishida S (2019). Galectin-1 promotes choroidal neovascularization and subretinal fibrosis mediated via epithelial-mesenchymal transition. FASEB J.

[3] Shu DY, Butcher E, Saint-Geniez M. EMT and EndMT: Emerging Roles in Age-Related Macular Degeneration. Int J Mol Sci. 2020;21(12):4271. 10.3390/ijms21124271. PMID: 32560057; PMCID: PMC7349630. 10.3390/ijms21124271 PMC7349630 32560057

[4] Rossato FA, Su Y, Mackey A, Ng YSE. Fibrotic changes and endothelial-to-mesenchymal transition promoted by VEGFR2 antagonism alter the therapeutic effects of VEGFA pathway blockage in a mouse model of choroidal neovascularization. Cells. 2020 Sep 9;9(9):2057. 10.3390/cells9092057. PMID: 32917003; PMCID: PMC7563259. 10.3390/cells9092057 PMC7563259 32917003

[5] Nagai N, Suzuki M, Uchida A, Kurihara T, Kamoshita M, Minami S (2016). Non-responsiveness to intravitreal aflibercept treatment in neovascular age-related macular degeneration: implications of serous pigment epithelial detachment. Sci Rep.

[6] Hwang JC, Del Priore LV, Freund KB, Chang S, Iranmanesh R (2011). Development of subretinal fibrosis after anti-VEGF treatment in neovascular age-related macular degeneration. Ophthalmic Surg Lasers Imaging.

[7] Daniel E, Toth CA, Grunwald JE, Jaffe GJ, Martin DF, Fine SL (2014). Risk of scar in the comparison of age-related macular degeneration treatments trials. Ophthalmology..

[8] Casalino G, Stevenson MR, Bandello F, Chakravarthy U (2018). Tomographic biomarkers predicting progression to fibrosis in treated Neovascular age-related macular degeneration: a multimodal imaging study. Ophthalmol Retina.

[9] Shimizu H, Yamada K, Suzumura A, Kataoka K, Takayama K, Sugimoto M (2020). Caveolin-1 promotes cellular senescence in exchange for blocking subretinal fibrosis in age-related macular degeneration. Invest Ophthalmol Vis Sci.

[10] Mettu PS, Allingham MJ, Cousins SW. Incomplete response to Anti-VEGF therapy in neovascular AMD: Exploring disease mechanisms and therapeutic opportunities. Prog Retin Eye Res. 2020:100906. 10.1016/j.preteyeres.2020.100906. Epub ahead of print. PMID: 33022379. 10.1016/j.preteyeres.2020.100906 PMC10368393 33022379

[11] Uder C, Bruckner S, Winkler S, Tautenhahn HM, Christ B (2018). Mammalian MSC from selected species: features and applications. Cytometry A.

[12] Zhao G, Ge Y, Zhang C, Zhang L, Xu J, Qi L (2020). Progress of mesenchymal stem cell-derived exosomes in tissue repair. Curr Pharm Des.

[13] Li D, Gong Y. A promising strategy for non-arteritic anterior ischemic optic neuropathy: intravitreal mesenchymal stem cell exosome. Curr Stem Cell Res Ther. 2020. 10.2174/1574888X15666200814121849. Epub ahead of print. PMID: 32798377. 10.2174/1574888X15666200814121849 32798377

[14] Vlassov AV, Magdaleno S, Setterquist R, Conrad R (2012). Exosomes: current knowledge of their composition, biological functions, and diagnostic and therapeutic potentials. Biochim Biophys Acta.

[15] Mathieu M, Martin-Jaular L, Lavieu G, Thery C (2019). Specificities of secretion and uptake of exosomes and other extracellular vesicles for cell-to-cell communication. Nat Cell Biol.

[16] Mead B, Amaral J, Tomarev S (2018). Mesenchymal stem cell-derived small extracellular vesicles promote neuroprotection in rodent models of glaucoma. Invest Ophthalmol Vis Sci.

[17] Ma M, Li B, Zhang M, Zhou L, Yang F, Ma F (2020). Therapeutic effects of mesenchymal stem cell-derived exosomes on retinal detachment. Exp Eye Res.

[18] Zhang W, Wang Y, Kong Y (2019). Exosomes derived from mesenchymal stem cells modulate miR-126 to ameliorate hyperglycemia-induced retinal inflammation via targeting HMGB1. Invest Ophthalmol Vis Sci.

[19] He GH, Zhang W, Ma YX, Yang J, Chen L, Song J (2018). Mesenchymal stem cells-derived exosomes ameliorate blue light stimulation in retinal pigment epithelium cells and retinal laser injury by VEGF-dependent mechanism. Int J Ophthalmol.

[20] Mead B, Ahmed Z, Tomarev S (2018). Mesenchymal stem cell-derived small extracellular vesicles promote neuroprotection in a genetic DBA/2J mouse model of glaucoma. Invest Ophthalmol Vis Sci.

[21] Yu B, Zhang X, Li X (2014). Exosomes derived from mesenchymal stem cells. Int J Mol Sci.

[22] Mathew B, Ravindran S, Liu X, Torres L, Chennakesavalu M, Huang CC (2019). Mesenchymal stem cell-derived extracellular vesicles and retinal ischemia-reperfusion. Biomaterials..

[23] Nuzzi R, Caselgrandi P, Vercelli A, Cantore S (2020). Effect of mesenchymal stem cell-derived exosomes on retinal injury: a review of current findings. Stem Cells Int.

[24] Yu B, Shao H, Su C, Jiang Y, Chen X, Bai L (2016). Exosomes derived from MSCs ameliorate retinal laser injury partially by inhibition of MCP-1. Sci Rep.

[25] Busser H, Najar M, Raicevic G, Pieters K, Velez Pombo R, Philippart P (2015). Isolation and characterization of human mesenchymal stromal cell subpopulations: comparison of bone marrow and adipose tissue. Stem Cells Dev.

[26] Connor TB, Roberts AB, Sporn MB, Danielpour D, Dart LL, Michels RG (1989). Correlation of fibrosis and transforming growth factor-beta type 2 levels in the eye. J Clin Invest.

[27] Kobayashi M, Tokuda K, Kobayashi Y, Yamashiro C, Uchi SH, Hatano M (2019). Suppression of epithelial-mesenchymal transition in retinal pigment epithelial cells by an MRTF-A inhibitor. Invest Ophthalmol Vis Sci.

[28] Zhang F, Ren CC, Liu L, Chen YN, Yang L, Zhang XA (2018). HOXC6 gene silencing inhibits epithelial-mesenchymal transition and cell viability through the TGF-beta/smad signaling pathway in cervical carcinoma cells. Cancer Cell Int.

[29] Li PD, Chen P, Peng X, Ma C, Zhang WJ, Dai XF (2018). HOXC6 predicts invasion and poor survival in hepatocellular carcinoma by driving epithelial-mesenchymal transition. Aging (Albany NY).

[30] You X, Zhou Z, Chen W, Wei X, Zhou H, Luo W (2020). MicroRNA-495 confers inhibitory effects on cancer stem cells in oral squamous cell carcinoma through the HOXC6-mediated TGF-beta signaling pathway. Stem Cell Res Ther.

[31] Ding SLS, Kumar S, Mok PL. Cellular reparative mechanisms of mesenchymal stem cells for retinal diseases. Int J Mol Sci. 2017;18(8):1406. 10.3390/ijms18081406. PMID: 28788088; PMCID: PMC5577990. 10.3390/ijms18081406 PMC5577990 28788088

[32] Mead B, Tomarev S (2017). Bone marrow-derived mesenchymal stem cells-derived exosomes promote survival of retinal ganglion cells through miRNA-dependent mechanisms. Stem Cells Transl Med.

[33] Kim JY, You YS, Kim SH, Kwon OW (2017). Epiretinal membrane formation after intravitreal autologous stem cell implantation in a retinitis pigmentosa patient. Retinal Cases Brief Rep.

[34] Huang H, Kolibabka M, Eshwaran R, Chatterjee A, Schlotterer A, Willer H (2019). Intravitreal injection of mesenchymal stem cells evokes retinal vascular damage in rats. FASEB J.

[35] Jonas S, Izaurralde E (2015). Towards a molecular understanding of microRNA-mediated gene silencing. Nat Rev Genet.

[36] Wang SS, Wang C, Chen H. MicroRNAs are critical in regulating smooth muscle cell mineralization and apoptosis during vascular calcification. J Cell Mol Med. 2020. 10.1111/jcmm.16005. Epub ahead of print. PMID: 33089928. 10.1111/jcmm.16005 PMC7754013 33089928

[37] Berber P, Grassmann F, Kiel C, Weber BH (2017). An eye on age-related macular degeneration: the role of microRNAs in disease pathology. Mol Diagn Ther.

[38] Ertekin S, Yildirim O, Dinc E, Ayaz L, Fidanci SB, Tamer L (2014). Evaluation of circulating miRNAs in wet age-related macular degeneration. Mol Vis.

[39] Li J, Hui L, Kang Q, Li R (2018). Down-regulation of microRNA-27b promotes retinal pigment epithelial cell proliferation and migration by targeting Nox2. Pathol Res Pract.

[40] Saika S (2006). TGFbeta pathobiology in the eye. Lab Investig.

[41] Chen CL, Chen YH, Tai MC, Liang CM, Lu DW, Chen JT (2017). Resveratrol inhibits transforming growth factor-beta2-induced epithelial-to-mesenchymal transition in human retinal pigment epithelial cells by suppressing the Smad pathway. Drug Des Devel Ther.

[42] Yu M, Zhan J, Zhang H (2020). HOX family transcription factors: related signaling pathways and post-translational modifications in cancer. Cell Signal.

[43] Shao J, Choudhary MM, Schachat AP (2016). Neovascular age-related macular degeneration. Dev Ophthalmol.

[44] Pegtel DM, Gould SJ (2019). Exosomes. Annu Rev Biochem.

